# Anti‐Hyperglycemic Effects of Oils and Extracts Derived from Sea Buckthorn – A Comprehensive Analysis Utilizing In Vitro and In Vivo Models

**DOI:** 10.1002/mnfr.202101133

**Published:** 2022-04-27

**Authors:** Nicole Ollinger, Cathrina Neuhauser, Bettina Schwarzinger, Melanie Wallner, Clemens Schwarzinger, Bernhard Blank‐Landeshammer, Roland Hager, Nadiia Sadova, Ivana Drotarova, Katrin Mathmann, Eugenia Karamouzi, Panagiotis Panopoulos, Gerald Rimbach, Kai Lüersen, Julian Weghuber, Clemens Röhrl

**Affiliations:** ^1^ FFoQSI – Austrian Competence Centre for Feed and Food Quality Safety & Innovation FFoQSI GmbH Technopark 1D Tulln 3430 Austria; ^2^ University of Applied Sciences Upper Austria Stelzhamerstrasse 23 Wels 4600 Austria; ^3^ Johannes Kepler University Institute for Chemical Technology of Organic Materials Linz 4040 Austria; ^4^ European Research & Development Rezos Brands 196 New National Road Patras‐Athens Patras 26443 Greece; ^5^ Institute of Human Nutrition and Food Science University of Kiel Hermann‐Rodewald‐Strasse 6 Kiel 24118 Germany

**Keywords:** diabetes, glucose metabolism, GLUT4, isorhamnetin, sea buckthorn

## Abstract

**Scope:**

Sea buckthorn (*Hippophaes rhamnoides*) is capable of ameliorating disturbed glucose metabolism in animal models and human subjects. Here, the effect of sea buckthorn oil as well as of extracts of fruits, leaves, and press cake on postprandial glucose metabolism is systematically investigated.

**Methods and results:**

Sea buckthorn did neither exert decisive effects in an in vitro model of intestinal glucose absorption nor did it alter insulin secretion. However, sea buckthorn stimulates GLUT4 translocation to the plasma membrane comparable to insulin, indicative of increased glucose clearance from the circulation. Isorhamnetin is identified in all sea buckthorn samples investigated and is biologically active in triggering GLUT4 cell surface localization. Consistently, sea buckthorn products lower circulating glucose by ≈10% in a chick embryo model. Moreover, sea buckthorn products fully revert hyperglycemia in the nematode *Caenorhabditis elegans* while they are ineffective in *Drosophila melanogaster* under euglycemic conditions.

**Conclusion:**

These data indicate that edible sea buckthorn products as well as by‐products are promising resources for hypoglycemic nutrient supplements that increase cellular glucose clearance into target tissues.

## Graphical abstract

Anti‐hyperglycemic activity of oils and extracts derives from sea buckthorn. Application of in vitro and in vivo models unravels key mechanisms, including an increase of GLUT4 translocation and the decrease of hyperglycemia and blood glucose levels.

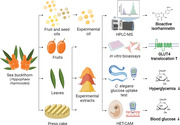

## 1 Introduction

Obesity is no longer an endemic metabolic disorder restricted to high‐income countries. Instead, overweight and obesity have become pandemic. Globally, more people are obese (BMI ≥ 30) than underweight (BMI ≤ 18.5) and the mean global BMI keeps continuously increasing linearly over time.^[^ ^1^ ^]^ Obesity, especially abdominal obesity, is the main inducer of disturbed glucose homeostasis, insulin resistance and ultimately diabetes mellitus, type 2. High fasting plasma glucose, an easily accessible parameter for disturbed glucose homeostasis, is among the top five risk factors for human health. Exposure to this risk factor is increasing and 6.62 million deaths were globally associated with high fasting plasma glucose in 2019.^[^ ^2^ ^]^

Lifestyle modification – foremost weight loss – is effective against early insulin resistance. However, pharmaceutical intervention becomes indispensable in many patients. In addition, intervention with natural nutrient supplements might be advantageous for selected individuals, for instance in combination with lifestyle modifications in early insulin resistance rendering pharmacological therapy unnecessary. Furthermore, natural products such as non‐flavonoid polyphenols, flavonoids, and others have been proven effective in supporting pharmaceutical intervention.^[^ ^3^ ^]^

Noteworthy, a considerable number of pharmaceuticals are derived from natural products. For instance, goat's rue (*Galega officinalis*) was discovered to contain galegine and used to treat diabetes in the 1920s. Based on this compound, metformin was developed which is still broadly used as a hypoglycemic drug.^[^ ^4^ ^]^ To date, various natural bioactive compounds and extracts were identified as effective hypoglycemic agents in human trials. These natural products include curcuminoids,^[^ ^5^ ^]^ green tea,^[^ ^6^ ^]^ guava,^[^ ^7^ ^]^ and many more.^[^ ^8^ ^]^ In addition, sea buckthorn (*Hippophae rhamnoides*) and its products are proposed hypoglycemic agents: several studies revealed amelioration of glucose tolerance and insulin resistance by different components of sea buckthorn in rodent models.^[^ ^9^, ^10^ ^]^ In human subjects, sea buckthorn fruits improved postprandial glycemic profile^[^ ^11^ ^]^ and application for five weeks led to decreased fasting plasma glucose levels in subjects with impaired glucose regulation.^[^ ^12^ ^]^ In contrast, no effect on fasting plasma glucose levels was observed in obese subjects not selected for impaired glucose regulation.^[^ ^13^ ^]^

Systemic glucose metabolism is influenced at various steps, most of which rely on delicately balanced regulatory mechanisms. Selected canonical postprandial processes include i) uptake of dietary glucose by the intestine via SGLT1; ii) concentration‐dependent uptake of glucose into the pancreas via GLUT2 followed by insulin secretion; and iii) insulin‐dependent uptake into muscle and adipose tissue via GLUT4. In this study, we focused our analyses on these three central steps in systemic postprandial glucose homeostasis. Of note, especially insulin secretion and glucose uptake into muscle and adipose tissue are impaired in early insulin resistance as well as in manifest type 2 diabetes mellitus.^[^ ^14^ ^]^

To comprehensively study the effect of sea buckthorn on postprandial glucose metabolism, we combined suitable cell culture model systems mimicking the intestinal barrier as well as cell models engineered to study insulin secretion and GLUT4 translocation. Moreover, in vivo model systems – specifically the chick embryo model and the non‐vertebrates *Caenorhabditis elegans* and *Drosophila melanogaster* – were utilized. These higher organisms provide the possibility for systemic metabolic analysis and are not subjected to ethical constraints.^[^ ^15^ ^]^

Additionally, this study aimed to compare the biological effects as well as the chemical composition of active ingredients in different plant parts and products of sea buckthorn including oil, fruits, leaves, and press cake extracts. This is because production of sea buckthorn oils requires harvesting of entire branches including leaves in order to obtain fruits and seeds. In addition, press cakes arise from oil production. Therefore, investigating the beneficial effects of these by‐products on human health was likewise covered by our study.

## 2 Results

### 2.1 Analytical Characterization of Sea Buckthorn Products

Initially, sea buckthorn products were characterized in terms of putative biologically active compounds. Sea buckthorn oil, leaves, fruits, and press cake were obtained from the same geographic region. In addition, two further kinds of sea buckthorn oil originating from different geographic regions were used. Ethanolic extracts were prepared from leaves, fruits, and press cake and were first subjected to chemical sum parameter analysis. Total polyphenolic content was ∼5‐fold and ∼10‐fold higher in leaf extracts compared to press cake and fruit extract, respectively (**Table** **1**). Anti‐oxidative capacity was similarly highest in leaf extracts followed by press cake and fruit extract. Determination of TPC and TEAC in oils was not feasible because their hydrophobicity hindered application in these assays.

**Table 1:** Analytical characterization of sea buckthorn products

	Oil A (Zagori)	Oil B (Meteora)	Oil C (Konitsa)	Leaf extract (Zagori)	Fruit extract (Zagori)	Press cake extract (Zagori)
TPC	–	–	–	2471.8	233.4	554.4
TEAC	–	–	–	93.6	4.8	18.1
Isorhamnetin	21.1	37.1	48.0	466.7	80.0	233.3
C14:0	0.39	0.25	0.39	–	–	–
C16:0	36.11	34.21	38.62	–	–	–
C16:1 n7	26.01	28.68	26.62	–	–	–
C18:0 C	1.04	0.98	1.07	–	–	–
C18:1 n9	26.24	25.01	23.96	–	–	–
C18:1 n7	6.57	7.49	5.99	–	–	–
C18:2 n6	3.03	2.81	2.68	–	–	–
C20:0	0.41	0.37	0.44	–	–	–
C20:1 n9	0.14	0.20	0.20	–	–	–
C22:0	0.21	n.d.	0.28	–	–	–
C24:0	0.25	n.d.	n.d.	–	–	–
Total SFA	38.10	35.81	40.62	–	–	–
Total MUFA	58.87	61.38	56.70	–	–	–
Total PUFA	3.03	2.81	2.68	–	–	–

All data represent means from technical triplicates. Fatty acid species are given as relative content [%]; SFA, MUFA, and PUFA. Isorhamnetin: [µg isorhamnetin per g oil] or [µg isorhamnetin per g dry mass] for extracts. MUFA, monounsaturated fatty acids; n.d., not detectable; PUFA, polyunsaturated fatty acids; SFA, saturated fatty acids; TEAC, trolox equivalent antioxidant capacity [mmol TE L^−1^]; TPC, total polyphenolic content [mg GAE L^−1^].

Next, in‐depth analyses of putative biologically active compounds was conducted in sea buckthorn products, foremost in sea buckthorn oil. HPLC‐MS analysis revealed particular high abundance of a single compound that was identified as isorhamnetin using authentic standards (Supporting Information Table S1 and Supporting Information Figure S1). In addition, several isorhamnetin glycosides as well as quercetin were identified.

Isorhamnetin content was then quantitated by HPLC in sea buckthorn oils. Concentrations measured ranged from 21.1 to 48.0 µg g^−1^ oil dependent on its geographic origin. In extracts prepared from sea buckthorn plant parts, isorhamnetin was most abundant in leaves followed by press cake and fruit extracts (Table 1). These concentrations correlated with TPC and TEAC measurements.

Analysis of fatty acid composition of sea buckthorn oils identified palmitic acid (C16:0) as the most abundant fatty acid, followed by palmitoleic acid (C16:1 n7) and oleic acid (C18:1 n9; Table 1). The relatively high content of palmitoleic acid (26.0%–28.7%) is characteristic for the fruits and pulp of sea buckthorn and in accordance with previous studies.^[^ ^16^ ^]^ In total, sea buckthorn oils consisted of 35.8%–40.6% saturated fatty acids, 56.7%–61.4% monounsaturated fatty acids and 2.8%–3.0% polyunsaturated fatty acids. No major differences were identified regarding geographic origin of the oils.

Given the similar results regarding the composition of sea buckthorn oils, only one of the oils was included in the majority of the following biological studies. Since the samples of fruits, leaves, and press cake originated from the geographic region of Zagori, oil A from the same region was chosen.

### 2.2 Effect of Sea Buckthorn Products on Glucose Absorption and Insulin Secretion

We aimed to comprehensively investigate the physiological effects of sea buckthorn products on fundamental steps in postprandial glucose metabolism, that is, intestinal glucose absorption, regulation of insulin secretion, and insulin‐dependent glucose uptake into target tissues.

**Figure 1:**
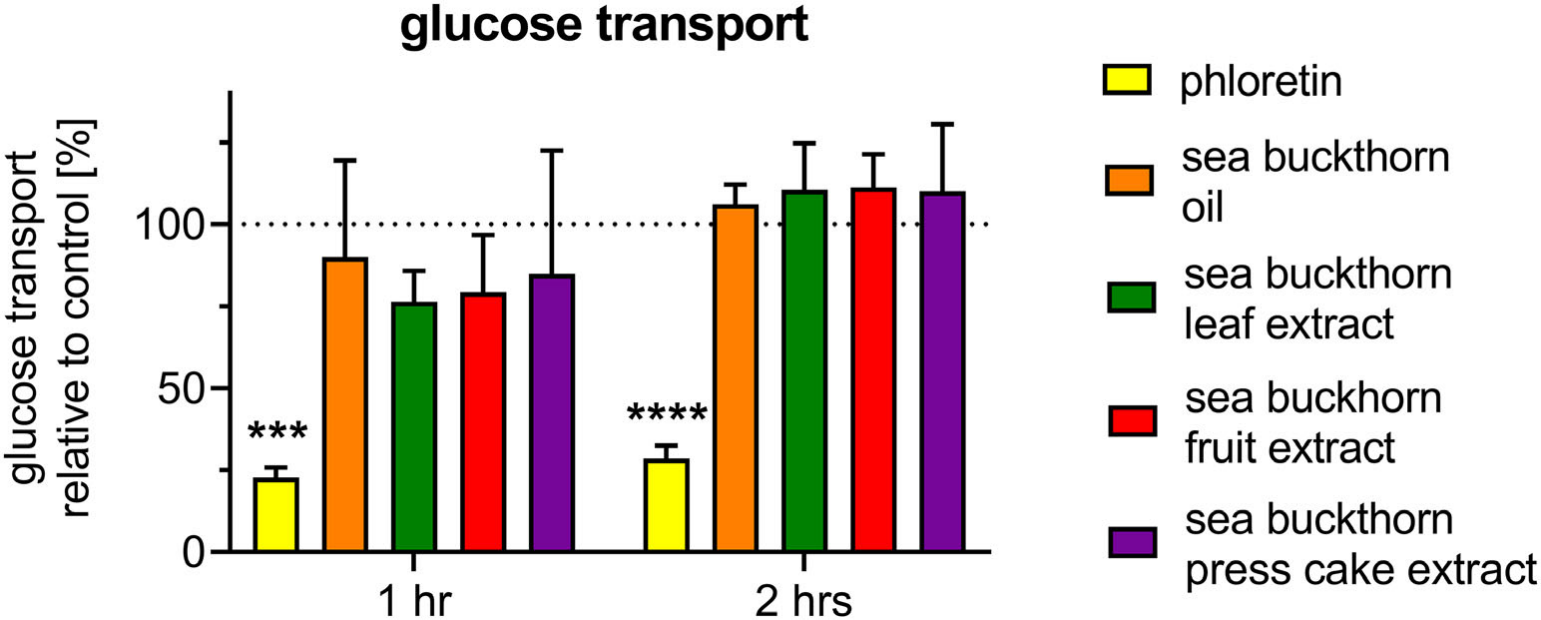
Effect of Sea buckthorn products on transepithelial glucose transport. Caco‐2 cells were cultivated on transwell inserts and differentiated as described in the methods section. Apical to basolateral glucose transport was measured in the presence of sea buckthorn oil (oil A, Zagori; 5 g L^−1^) or extracts (50 mg/L). Basolateral glucose concentrations were measured after the indicated time points by HPLC. TEER values and absence of xylitol transport were assessed to ensure cell layer integrity. Phloretin, an established SGLT1 inhibitor, served as positive control. Data represent mean ± SD (*n* = 3). SD, standard deviation; TEER, transepithelial electrical resistance.

Polarized human Caco‐2 cells cultivated in 3D on transwell inserts were utilized as a model system to mimic intestinal glucose absorption. As expected, glucose absorption was effectively inhibited by phloretin, an established inhibitor of SGLT1 (**Figure** **1**) throughout the time points measured. However, glucose transport was not decisively altered by sea buckthorn products.

**Figure 2:**
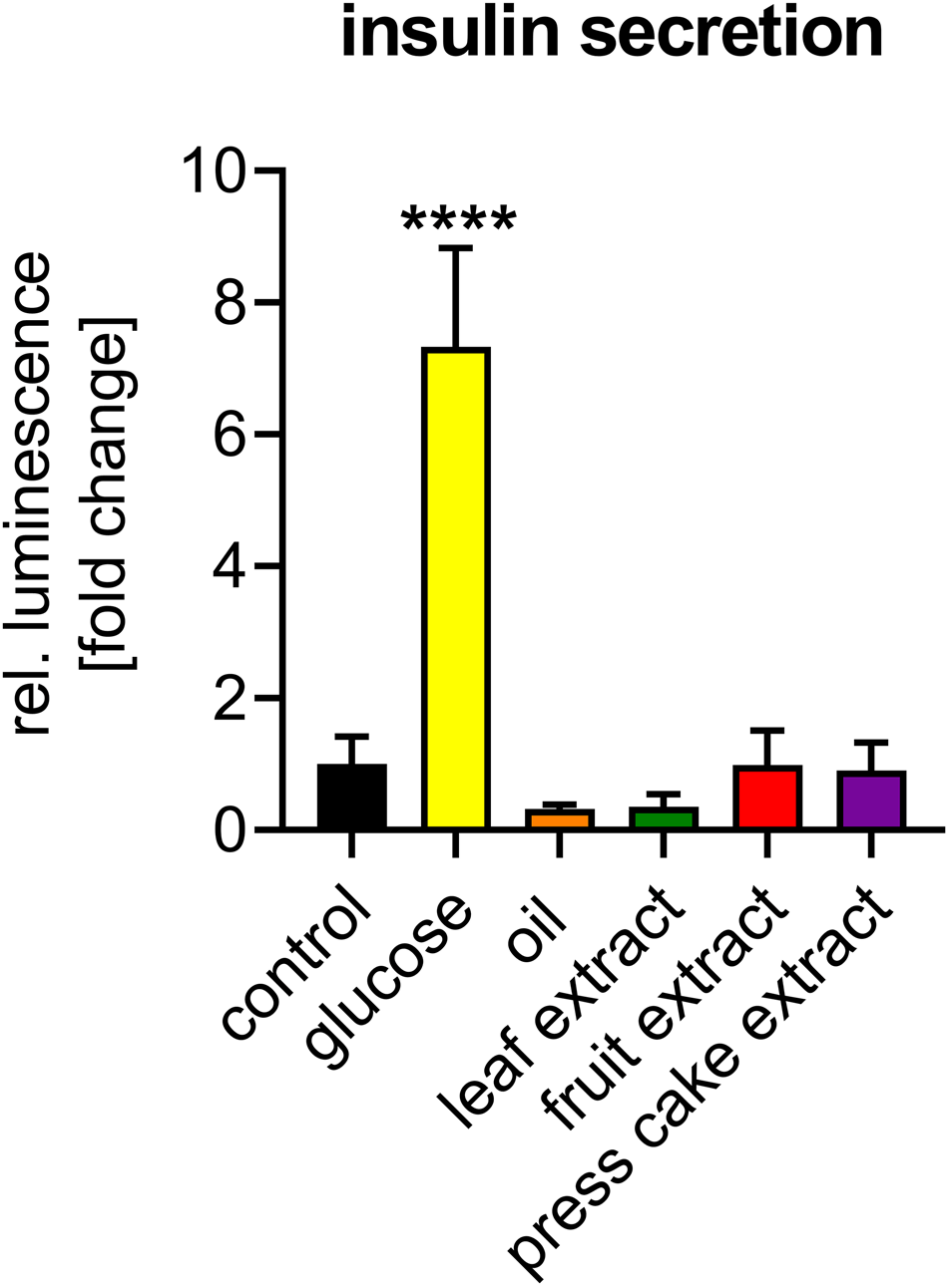
Sea buckthorn does not modulate insulin secretion. MIN6 β‐cells expressing luciferase‐tagged insulin were stimulated with sea buckthorn oil (oil A, Zagori; 5 g L^−1^) or extracts (50 mg L^−1^) in the absence of glucose for 1 h. Insulin secretion was assessed by luminescence. 10 mM glucose served as positive control. Data represent mean ± standard deviation (SD) (*n* = 4).

Next, putative effects of sea buckthorn products on insulin secretion were studied. MIN6 β‐cells expressing luciferase‐tagged insulin were utilized. The addition of sea buckthorn oil, leaf extract, fruit extract, and press cake at different concentrations displayed no modulating effect on the secretion of insulin (**Figure** **2**), while addition of glucose as a physiologically relevant positive control augmented insulin secretion considerably.

### 2.3 Sea Buckthorn Products Trigger GLUT4 Translocation

**Figure 3:**
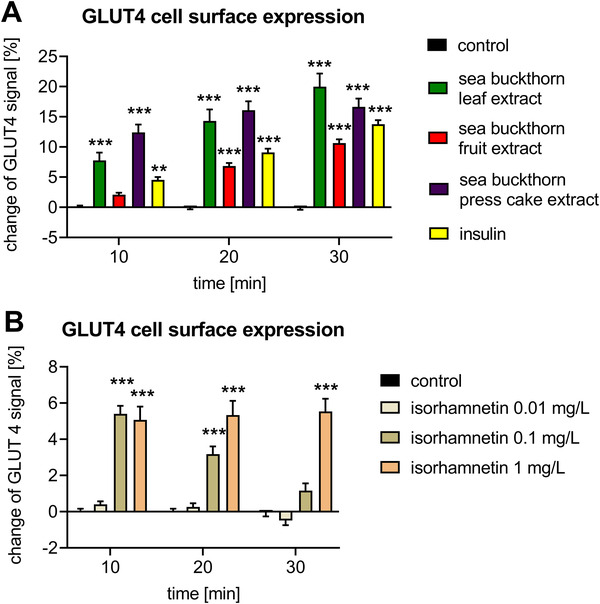
Sea buckthorn increases GLUT4 cell surface localization. GLUT4‐GFP translocation to the membrane was assessed by TIRF microscopy. (A) Effect of sea buckthorn extracts (1 mg L^−1^), insulin (100 nM) served as positive control. (B) Effects of isorhamnetin. Data represent mean ± SEM (*n* = 36–48 cells per condition). SEM, standard error of the mean; TIRF, total internal reflection fluorescence.

Insulin‐mediated uptake of glucose by GLUT4 into muscle and adipose tissue represent the main mechanism of postprandial glucose clearance. Insulin thereby triggers translocation of GLUT4 to the plasma membrane for subsequent glucose uptake. Therefore, GLUT4 cell surface localization was assessed using TIRF microscopy allowing for selective excitation of fluorescently tagged GLUT4 at the plasma membrane. As expected, insulin triggered GLUT4 cell surface expression (**Figure** **3**A). Comparably, all sea buckthorn extracts tested increased GLUT4 cell surface localization in a time‐dependent manner (Figure 3A and Supporting Information Figure S2 for representative images). Unfortunately, treatment of cells with the oil resulted in high fluorescence background levels which hindered application of this method for sea buckthorn oil. Given the high abundance of isorhamnetin in sea buckthorn products, we investigated, if isorhamnetin is biologically active in triggering GLUT4 cells surface expression. Indeed, isorhamnetin alone augmented cell surface expression of GLUT4. While this effect was transient with 0.1 mg L^−1^ isorhamnetin, a higher concentration of 1 mg L^−1^ led to stable increases over time (Figure 3B). These results indicate that sea buckthorn extracts are capable of inducing GLUT4 cell surface expression comparable to insulin and that isorhamnetin is a main biologically active compound.

### 2.4 Potent Glucose‐Lowering Effect of Sea Buckthorn in Chick Embryo Model

**Figure 4:**
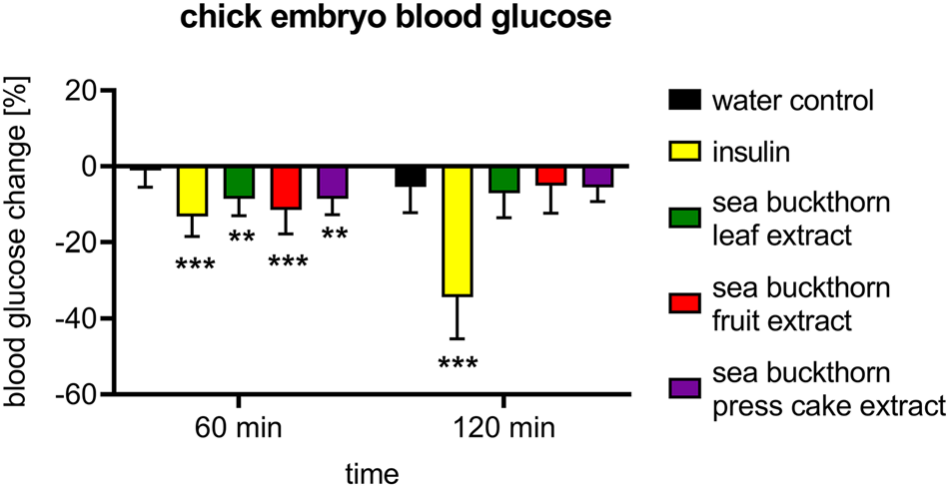
Sea buckthorn lowers systemic blood glucose in the chick embryo model. In ovo test for glucose inhibition in chick embryo. Sea buckthorn extracts (600 mg L^−1^) were applied to the chorioallantoic membrane and blood glucose levels were measured after the indicated time points. An insulin analog served as positive control. Data represent mean ± standard deviation (SD) (*n* = 10 eggs per condition and time point).

In order to test systemic glucose‐lowering effects of sea buckthorn in an intact organism without ethical constraints, the chick embryo model was utilized. At the developmental stage tested, chick embryos do not yet produce insulin on their own, but are sensitive to insulin. This model system is thus capable of reflecting insulin‐mediated glucose lowering effects as well as the effects of insulin‐mimicking plant extracts.^[^ ^17^ ^]^ Sea buckthorn oil did not diffuse through the chorioallantoic membrane and its effect could therefore not be assessed with this model system. However, application of extracts of sea buckthorn leaves, fruits, or press cake lowered systemic embryonic glucose levels comparable to insulin by approximately 10% after 60 min (**Figure** **4**). The glucose‐lowering capability of insulin was more pronounced after 120 min, while the effects of sea buckthorn extracts were transient.

### 2.5 Sea Buckthorn Products Revert Hyperglycemia in *C. elegans*

**Figure 5:**
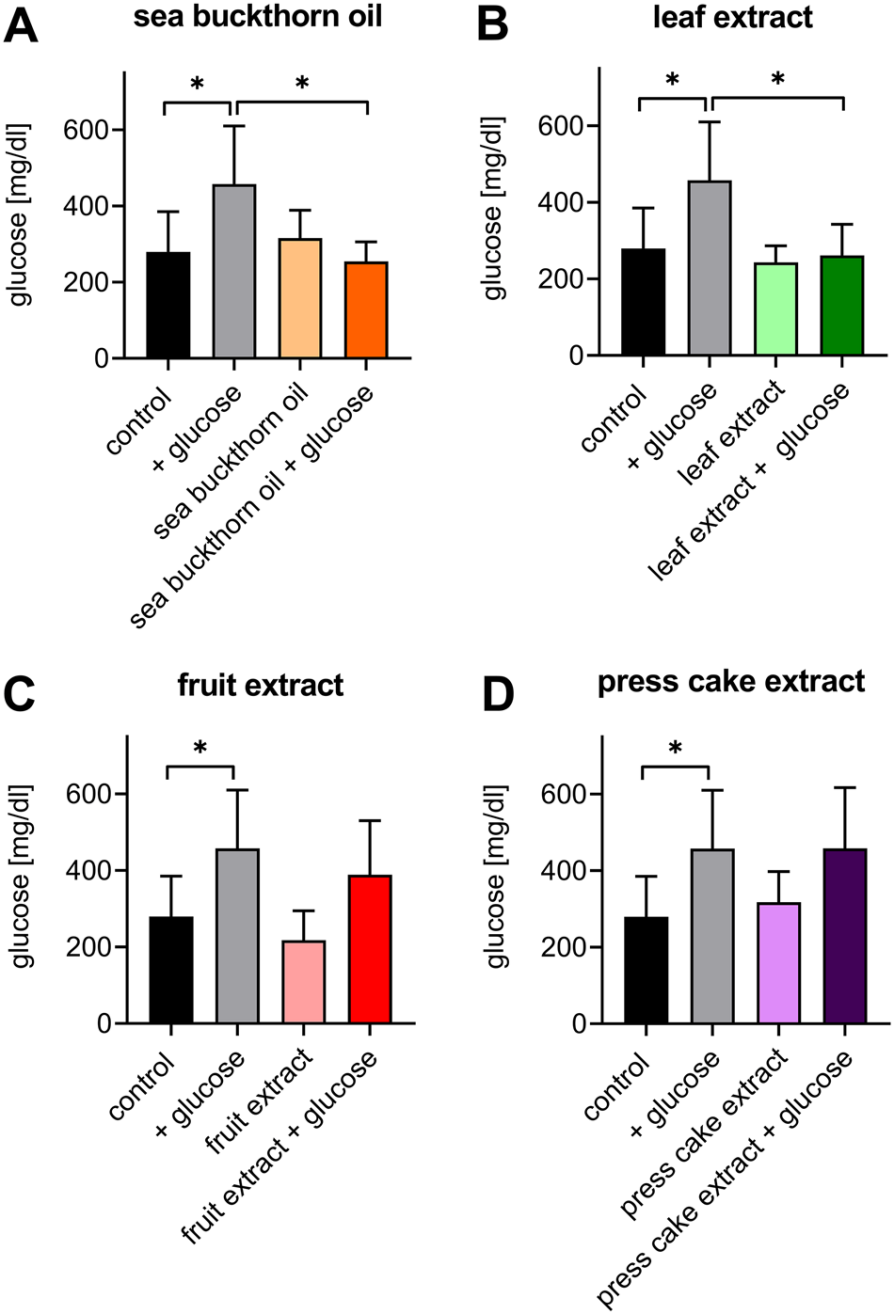
Sea buckthorn reverts hyperglycemia in *C. elegans*. Young adult *C. elegans* were transferred to NGM agar supplemented with 0 or 50 mM glucose with or without sea buckthorn oil or extracts. Nematodes were collected after 24 h and glucose concentration was measured. Glucose concentration after treatment with sea buckthorn oil (oil A, Zagori; 5 g L^−1^; A), sea buckthorn leaf extract (50 mg L^−1^; B), sea buckthorn berries (50 mg L^−1^; C), and sea buckthorn press cakes (50 mg L^−1^; D). Data represent mean ± standard deviation (SD) (*n* ≥ 4).

To test effects on glucose metabolism in an independent, non‐vertebrate model system, sea buckthorn oil and extracts of leaves, fruits, and press cake were tested in the nematode *C. elegans*. Addition of glucose to the agar resulted in considerably increased systemic glucose levels in the nematode after 24 h. Parallel application of 0.5% sea buckthorn oil entirely reverted this hyperglycemic effect (**Figure** **5**A). Decreased glucose levels were not due to toxic adverse effects, since sea buckthorn oil concentrations up to 2.5% did not result in decreased *C. elegans*’ viability (data not shown). Comparably, systemic hyperglycemia was fully reverted by treatment with sea buckthorn leave extracts (Figure 5B). Moreover, systemic glucose levels tended to be lower in the presence of sea buckthorn fruit extracts in combination with glucose. In contrast, press cake extracts were inactive (Figure 5C, D).

### 2.6 Sea Buckthorn Products Do Not Alter Systemic Glucose Levels in *D. melanogaster* on Standard Diet

**Figure 6:**
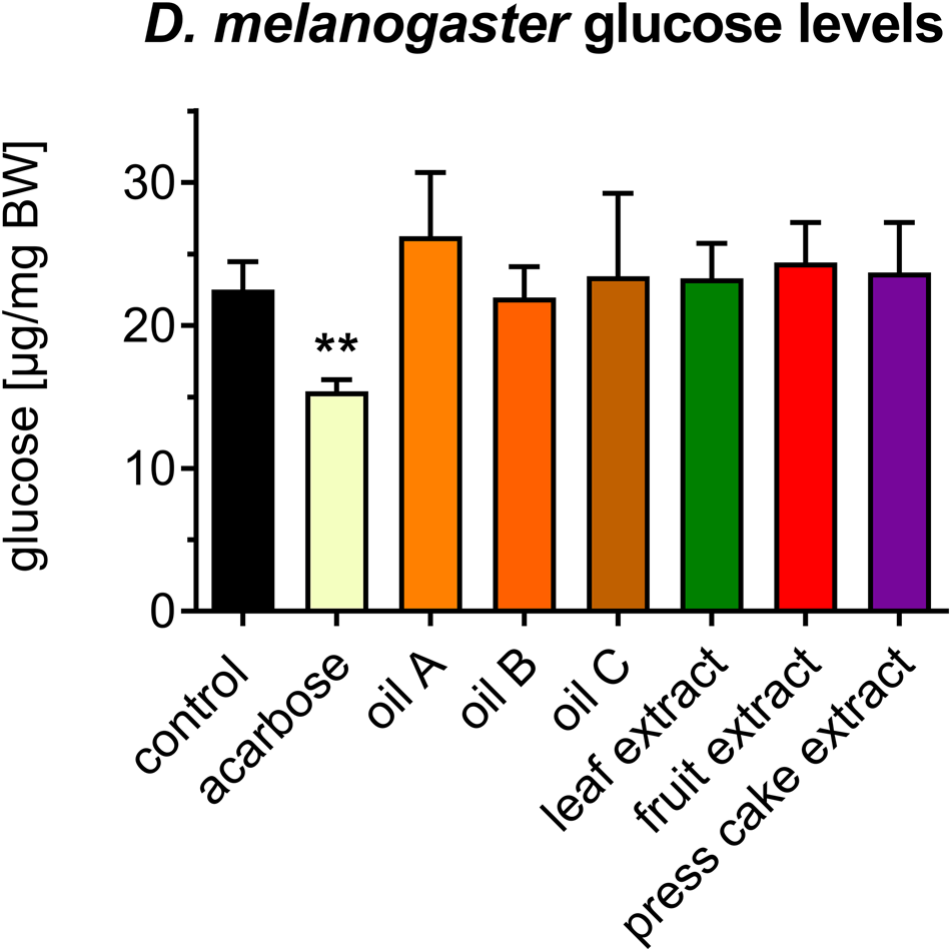
Sea buckthorn products do not alter systemic glucose in *D. melanogaster*. Animals were kept on standard diet in the presence or absence of sea buckthorn oils or extracts for 7 days. Glucose was determined in whole fly lysates and normalized to body weight. The hypoglycemic agent acarbose served as positive control. Data represent mean ± standard deviation (SD) (*n* = 6 groups of 10 flies each).

Finally, the effect of sea buckthorn was investigated in *D. melanogaster*, a model organism for nutritional research displaying high homology to humans in terms of metabolic pathways.^[^ ^18^ ^]^ Feeding trials were performed for 7 days and the anti‐diabetic drug acarbose, an inhibitor of carbohydrate digestion, was used as positive control. None of the experimental diets altered body weights (data not shown). Only acarbose reduced systemic glucose levels significantly (**Figure** **6**), while sea buckthorn oils and extracts were ineffective. These data indicate that sea buckthorn products revert hyperglycemia in *C. elegans*, but do not reduce systemic glucose levels in *D. melanogaster* on standard diet.

## 3 Discussion

Here, we provide a comprehensive analysis of the effect of sea buckthorn on glucose homeostasis on central regulatory steps of postprandial glucose metabolism. Various biological models including human cells lines, non‐vertebrate organisms, and the chick embryo model were utilized as complementary systems.

Sea buckthorn is a rich source of biologically active compounds suitable to exert beneficial effects to human health. In particular, sea buckthorn oil contains ∼190 compounds with putative biological activity.^[^ ^19^ ^]^ HLPC‐MS analyses in combination with biological assays revealed that isorhamnetin is highly abundant in all sea buckthorn products tested and is one of the biologically active compounds triggering GLUT4 cell surface localization. This finding is consistent with previous reports which showed that isorhamnetin promotes glucose uptake in myotubes.^[^ ^20^ ^]^ Noteworthy, the effect of sea buckthorn products on GLUT4 translocation exceeded the effects observed after treatment with isolated isorhamnetin. This suggests the presence of additional biologically active compounds that synergize with isorhamnetin. Indeed, gallic acid, which is abundant in sea buckthorn leaves, likewise induces GLUT4 cell surface localization.^[^ ^21^ ^]^

As we observed no effects of sea buckthorn oils and extracts on glucose absorption and no effect on insulin secretion, we hypothesize that the main glucose‐lowering property of sea buckthorn is increased insulin‐mediated glucose clearance by muscle and adipose tissue. This is consistent with the fact that sea buckthorn lowered glucose levels in the chick embryo model. The advantage of this model system is its sensibility to insulin, while no endogenous insulin is detected until day 12 of development.^[^ ^22^ ^]^ The use of the chick embryo model on day 11 therefore enables the direct examination of glucose clearance from the circulation without interference of endogenous insulin production. A putative limitation of this model system is the lack of a GLUT4 homologue,^[^ ^23^ ^]^ suggesting that another, insulin‐responsive glucose uptake mechanism is present in chick embryo.

In overweight human subjects, consumption of a sea buckthorn‐rich meal led to decreased and delayed insulin response.^[^ ^11^ ^]^ The authors attribute this effect to improved insulin sensitivity that in turn allowed for decreased insulin production. This suggests that sea buckthorn products are capable of increasing clearance of glucose from the bloodstream into muscle and adipose tissue allowing for reduced insulin production and pancreatic β cell protection.

A further finding of our study is that sea buckthorn is able to revert hyperglycemia in the nematode *C. elegans*. Glucose concentrations resembling the hyperglycemic conditions in diabetic patients can be easily achieved in *C. elegans*.^[^ ^24^ ^]^ Comparable to humans, *C. elegans* can store excess glucose as glycogen or fatty acids after de novo lipogenesis. In addition, C. *elegans* is capable of storing excess glucose as the trisaccharide trehalose.^[^ ^25^ ^]^ Importantly, the insulin signaling pathway is a conserved regulator of glucose storage across species including humans and nematodes. In particular, daf‐2, an insulin receptor‐like gene, controls lipid and carbohydrate storage in *C. elegans*.^[^ ^26^ ^]^ We therefore hypothesize that the observed anti‐hyperglycemic effect of sea buckthorn is due to increased cellular uptake followed by conversion and storage as glycogen, trehalose or triglycerides. Of note, sea buckthorn products counteracted hyperglycemia, but did not lower glucose levels under basal conditions. A potential limitation of using *C. elegans* is differential regulation of FTG‐1, the only identified GLUT homologue in the nematode. Despite the fact that FTG‐1‐mediated glucose uptake is regulated by insulin signaling, its function is regulated by glycosylation rather than by translocation as it is the case for GLUT4.^[^ ^27^ ^]^

While hyperglycemia was effectively ameliorated by sea buckthorn products in *C. elegans*, neither sea buckthorn oil nor extracts lowered systemic glucose levels in *D. melanogaster*. Drosophila expresses several functional insulin‐like peptides.^[^ ^18^ ^]^ While a GLUT4 homolog remains to be identified, insulin was shown to increase the function of transgenic GLUT4 in drosophila fat cells, indicating conserved glucose uptake processes.^[^ ^28^ ^]^ Noteworthy, these experiments were conducted under euglycemic standard conditions based on feed containing 10% starch, which is a putative limitation. This is because trehalose, but not glucose, is the main carbohydrate in drosophila's circulation under euglycemic conditions.^[^ ^29^ ^]^ Indeed, in previous studies a high carbohydrate diet containing sucrose concentration of 1 mol L^−1^ (equivalent to ∼34% sucrose) was used to induced insulin resistance in drosophila.^[^ ^30^ ^]^

Besides studying physiological effects of sea buckthorn products, this study aimed to compare diverse sea buckthorn products and plant parts in terms of their bioactive compounds and biological effects. Harvesting sea buckthorn berries is laborious and is often accomplished by removal of complete branches followed by separation of the berries. Therefore, considerable amounts of by‐products including leaves emerge. Similarly, press cakes originate from oil production which are normally not further utilized. Therefore, using different plant parts and remnants of sea buckthorn for the subsequent production of dietary supplements is a sustainable approach. While the different matrices of oils and aqueous extracts impeded direct comparison in some of the analysis performed, the presence of isorhamnetin was clearly confirmed in all sea buckthorn products tested. Furthermore, biological effects were largely comparable throughout the biological assays performed. Therefore, sea buckthorn products that beneficially affect human health are not necessarily restricted to the use of oils and fruits.

Taken together, we have identified sea buckthorn oils and fruits as well as leaves and press cake as promising raw materials for the generation of nutrient supplement to counteract dysregulated glucose metabolism by increasing insulin‐dependent uptake of glucose into target tissues.

## 4 Experimental Section

### Sea Buckthorn Samples, Sample Preparation, and Extracts

Sea buckthorn (*Hippophaes rhamnoides*) products were kind gifts from REZOS BRANDS S.A. (Patras, Greece). Sea buckthorn leaves, fruits, and press cake were derived from the region of Zagori (Greece). Sea buckthorn oil A originated from the same geographic region; in addition, oils from two different geographic regions (oil B: Meteora, Greece; oil C: and Konitsa, Greece) were included.

Sea buckthorn leaves, dried fruits, or press cake were used for extract preparation as follows: Six g of frozen leaves, dried fruits or press cake were mixed with 25 g of ethanol (50%) followed by sonication for 1 h. Afterwards, suspensions were incubated in a uniTHERMIX 2 thermoshaker (LLG Labware, Meckenheim, Germany) at 400 rpm at 50°C for 24 h. Suspensions were centrifuged (6000x*g*, 10 min, RT) and the supernatant was used for dry matter determination utilizing a Sartorius Moisture Analyzer (Sartorius AG, Goettingen, Germany). Extracts were diluted with 50% ethanol to stock solutions of 10 g L^−1^ dry matter. These stock solutions were further diluted 1/200 to 1/1000 in respective assay buffers for cell culture experiments (see below).

For cell culture experiments, oils were solubilized in respective assay buffers at a concentration of 5% w/v by several cycles of vigorous vortexing and incubation in an ultrasonic bath for a total of 45 min. Resulting homogenous solutions were stable for at least 24 h. Solubilized oils were utilized for experiments immediately and diluted 1/10 to final concentrations in respective assay buffers.

### Chemical Sum Parameters

Chemical sum parameters were determined from extracts from fruits, leaves or press cake stock solutions (10 g L^−1^). Total phenolic content (TPC) were measured as described.^[^ ^31^ ^]^ Gallic acid was used as standard and total phenolics were expressed as mg gallic acid equivalents per liter (mg GAE/L). Trolox equivalent antioxidant capacity (TEAC) was quantitated using the ABTS decolorization assay.^[^ ^32^ ^]^ 6‐Hydroxy‐2,5,7,8‐tetramethylchroman‐2‐carboxylic acid (trolox) was used as standard and results were expressed as mmol trolox equivalents per liter (mmol TE/L).

### HPLC‐MS and HPLC

For HPLC analyses, 3 g of oil were extracted with 1.5 mL methanol by vigorous vortexing followed by incubation in an ultrasonic water bath for 5 min. Samples were centrifuged (4000x*g*, 1 min, RT) for phase separation. Extraction was repeated twice, pooled methanolic phases were evaporated to dryness and residues were resuspended in acetonitrile for subsequent HPLC analyses. Stock solutions of leave, fruit, and press cake extracts were directly subjected to HPLC analysis.

HPLC‐MS analyses were performed by reversed‐phase chromatography using a Surveyor HPLC (Thermo Fisher Scientific) equipped with an Accucore C18 column (150 mm × 3.0 mm i.d., 2.6 µm particle size; Thermo Fisher Scientific) as described.^[^ ^33^ ^]^ High‐resolution mass spectra were obtained using an LTQ Orbitrap Velios (Thermo Fisher Scientific) with an APCI source operated in positive and negative ionization mode. The resolution was set to 30 000 and diisooctylphthalate (*m*/*z* = 391.2843) was used as an internal standard for mass calibration. Spectra were collected from 80 to 1000 *m*/*z* and MS2 spectra were automatically recorded from the most intense peaks. Data were analyzed using Xcalibur (Thermo Fisher Scientific; version 2.2 SP1.48).

Absolute quantification of isorhamnetin was performed by HPLC on a Thermo Ultimate 3000 (Thermo Fischer Scientific) using an identical chromatographic procedure as described above. Compounds were detected by an UV detector (260 nm; Thermo Fischer Scientific). Isorhamnetin (Extrasynthese, Genay Cedex, France) was used as standard for calibration.

### Fatty Acid Methyl Ester (FAME) Measurements

A two‐step transesterification method was performed according to ISO 12966‐2:2017 with slight modifications. Briefly, aliquots of the oils were mixed with 200 µL 0.2 M sodium methoxide (Alfa Aesar, Thermo Fisher Scientific, Heysham, UK) and incubated at 60°C for 45 min under shaking. After the addition of 70 µL of 1 M methanolic sulfuric acid, samples were incubated a further 30 min at 60°C. FAMEs were extracted by addition of 600 µL saturated sodium chloride and 500 µL *n*‐hexane (Suprasolv, Merck KGaA, Darmstadt, Germany). Pentadecanoic acid (Sigma–Aldrich, St. Louis, MO, USA) was used as a recovery standard. GC‐MS analysis was performed as described previously.^[^ ^34^ ^]^ Full scans from *m*/*z* 40 to 400 were recorded and SIM scans at *m*/*z* 55, 67, 74, and 79 were used for quantification with the Supelco 37 Component FAME Mix (Sigma–Aldrich, St. Louis, MO, USA) as an external standard.

### Glucose Transport Assay

For glucose transport assays, human Caco‐2 cells (purchased from DSMZ, Braunschweig, Germany) were cultivated under standard conditions in minimum essential medium with Earle's salts supplemented with 10% fetal bovine serum (FBS), 100 µg mL^−1^ penicillin/streptomycin, and 0.1% 2‐mercaptoethanol. For transport studies, cells were seeded at 5.6 × 10^5^ cells per transwell insert and cells were differentiated using Entero‐STIM Intestinal Epithelium Differentiation Medium (Corning, Wiesbaden, Germany) supplemented with 1% penicillin/streptomycin and 0.1% MITO+ Serum Extender (Corning).^[^ ^35^ ^]^ Monolayer integrity was assessed by transepithelial electrical resistance (TEER) measurement. For experiments, differentiated cells were washed twice with HEPES buffer. Five hundred µL cell culture medium supplemented with glucose (total glucose concentration: 13.5 g L^−1^) and xylitol (1.0 g L^−1^) were added to the apical compartment, while the basolateral compartment contained 800 µL of HEPES buffer. Test solutions were applied to the apical compartment at the indicated dilutions. Phloretin (100 mg L^−1^; Extrasynthese) was used as positive control. Samples of the basolateral compartment were taken at respective time points and analyzed for their glucose and xylitol content using HPLC analysis.^[^ ^35^ ^]^ Xylitol, which is not transported through intact cell layers, as well as TEER values, were used to monitor monolayer integrity throughout experiments.

### Insulin Secretion

Pancreatic MIN6 β cells expressing an insulin–Gaussia luciferase (Ins‐GLuc) biosensor were generated by stable expression of human insulin with Gaussia luciferase inserted into the C‐peptide sequence.^[^ ^36^ ^]^ Cells were a kind gift from Michael A. Kalwat (UT Southwestern Medical Center, Dallas, TX, USA).

Cells were cultured in Dulbecco's modified Eagle's medium (DMEM; PAN‐Biotech, Aidenbach, Germany) supplemented with 15% FBS, 1% penicillin/streptomycin, 0.5% G418, and 0.1% 2–mercaptoethanol at 37°C under standard conditions. For insulin secretion experiments, cells were seeded in 96‐well plates at 5 × 10^4^ cells per well and incubated for 3 days. Cells were washed twice with KRPH buffer and starved from glucose in KRPH buffer for 1 h. Afterwards, cells were again washed with KRPH buffer and incubated with the indicated concentrations of sea buckthorn oil preparations or extracts for another hour. 10 mM glucose served as positive control. Insulin secretion was measured by luminescence as described.^[^ ^37^ ^]^

### GLUT4 Cell Surface Localization

GLUT4 cell surface localization was measured by total internal reflection fluorescence (TIRF) microscopy allowing for selective excitation of membrane‐adjacent fluorescently tagged GLUT4 as previously described.^[^ ^38^ ^]^ Briefly, HeLa GLUT4‐myc‐GFP cells were grown in RPMI 1640 in 96‐well imaging plates (4 × 10^4^ cells per well) overnight. Cell culture medium was removed and, after washing the cells with Hank's balanced salt solution (Thermo Fisher, Waltham, MA, USA), replaced by the same for 3 h. The cells were incubated with insulin (100 nM) or sea buckthorn extracts (1 mg L^−1^) dissolved in KRPH buffer and imaged on an epi‐fluorescent Nikon Eclipse Ti2 microscope in objective‐type total internal reflection configuration via an 60× oil immersion objective (NA = 1.49, APON 60XO TIRF) as described previously.^[^ ^38^ ^]^ Background signals were subtracted and images were analyzed using the SPOTTY software package, which can be retrieved online: https://bioinformatics.fh‐hagenberg.at/site/fileadmin/user_upload/img_upload/projects/spotty.html. Application of sea buckthorn oil led to high signal background due to autofluorescence and data were not interpretable.

### In Ovo Blood Glucose Reduction

For systemic analysis of blood‐glucose modulation by sea buckthorn extracts an established chick embryo model was utilized.^[^ ^39^ ^]^ Fertilized hens’ eggs (derived from Lohmann classic brown chicken) were obtained freshly from a local breeder and incubated at 38°C with an average humidity of 40%–60% for 11 days. The eggshell was perforated with a pointed pair of tweezers in the air bladder area and 300 µL of fruit extract, leave extract, or press cake extract (final concentration: 600 mg L^−1^ in water) were injected. Water or an insulin analog (NovoRapid, 3 U mL^−1^) was used as negative or positive controls, respectively. Embryos’ blood glucose levels were determined after 1 and 2 h as described.^[^ ^39^ ^]^

### *Caenorhabditis elegans* Glucose Uptake Test

*C. elegans’* wild type strain N2 Bristol was obtained from the *C. elegans* Genetics Center (CGC, University of Minnesota, USA) and cultured on Nematode Growth Media (NGM) plates with OP50 *Escherichia coli* as food source at 20°C according to standard methods.^[^ ^40^ ^]^ Worms were synchronized as previously described.^[^ ^41^ ^]^

Glucose uptake was measured according to the protocol by Schlotter et al.^[^ ^24^ ^]^ with minor modifications. A total of 250 L4 worms were seeded into 6 cm petri dishes containing 12 mL of NGM agar containing 0 mM or 50 mM glucose and/or respective amounts of sea buckthorn extract or oil. After cultivation at 20°C for 24 h, worms were rinsed off the plate and transferred into 1.5 mL reaction vessels. Co‐extracted glucose from the petri dish and oil residues were removed by three washing steps with pure water. Worm pellets were dissolved in 20 µL water and stored at −20°C until further use. After thawing, worms were disrupted by sonication for 30 min, pelleted (16 000x*g*, 5 min, RT) and the remaining water was evaporated in a drying chamber at 80°C. Pellets were resuspended in 10 µL pure water and glucose was determined by Accu‐check performa (F. Hoffmann‐La Roche AG, Basel, Switzerland) with Accu‐check Inform II strips (F. Hoffmann‐La Roche AG, Basel, Switzerland).

For toxicity testing, seven concentrations (5–35 g L^−1^) of sea buckthorn oil were administered to young adult wild‐type worms in 12 well plates (100 worms per plate) in presence of OP50. After 24 h worms were collected and counted under a dissecting microscope (Olympus SZX16). Worms were scored as dead when physical stimulus by a silver wire failed to generate any response. The experiment was performed in triplicate.

### Drosophila Feeding Trials

Stocks of the *D. melanogaster* wild type strain w^1118^ (Bloomington Drosophila Stock Center #5905, Indiana University, Bloomington, USA) were maintained in climate cabinets (HPP750 or HPP110, Memmert, Schwabach, Germany) at 25°C, 60% humidity, and a 12/12 h light/dark cycle as previously described.^[^ ^42^ ^]^ Stocks were fed on Caltech medium consisting of 5.5% dextrose, 3.0% sucrose, 6.0% cornmeal, 2.5% inactive dry yeast (Kisker Biotech, Steinfurt, Germany), 1.0% agar Type II (Kisker, Steinfurt, Germany) with 0.15% Tegosept (Genesee Scientific, San Diego, USA), and 0.3% propionic acid (Carl Roth, Karlsruhe, Germany) serving as preservatives. For feeding assays, the starch‐based control food consisted of 10% soluble starch (VWR, Darmstadt, Germany), 4% inactive dry yeast, 1% agar, 0.3% propionic acid, and 0.15% Tegosept. Experimental diets were supplemented with sea buckthorn products at a concentration of 0.5% v/v or 1.8 µg mL^−1^ acarbose. Drosophila eggs were collected 3–4 h after laying, transferred into respective vials (∼100 eggs per vial) containing the different experimental diets and cultured under standard conditions. After larval development, pupation, and eclosion, the adult flies were synchronized and mated for 2 days. On day 3 after eclosion, mated female flies were sorted and further maintained by transferring them to fresh media every other day. On day 7, the flies were starved for 1.5 h to ensure an empty intestine, before they were anesthetized, counted, and weighed. Ten flies per treatment group were homogenized in PBS containing 0.05% Triton X100 for 10 min at 4°C and 25 Hz using a tissue lyser (Qiagen TissueLyser II, Hilden, Germany). Glucose levels were determined in whole fly lysates by employing the D‐Glucose‐HK assay kit (Megazyme, Wicklow, Ireland) and normalized to body weight.^[^ ^42^ ^]^

### Statistical Analysis

Data were analyzed via ANOVA followed by Dunnett's correction using GraphPad Prism (v8.4.3; GraphPad Software, San Diego, CA). Results were independently confirmed using Jupyter Notebook (v6.3.0) with the package Pingouin (v0.3.12). Significance is indicated as *p* < 0.5 (*), *p* < 0.01 (**), or *p* < 0.01 (***).

## Conflict of Interest

The authors declare no conflict of interest.

## Author Contributions

N.O: Conceptualization, methodology for nematode glucose test, investigation, data curation, writing – original draft preparation, and formal analyses. C.N, B.S., M.W., C.S., B.B‐L., R.H., and I.D.: Investigation. N.S.: Conceptualization, writing: reviewing and editing. K.M.: Formal analyses. E.K.: Conceptualization, writing – reviewing and editing. P.P.: Conceptualization, writing – reviewing and editing. G.R.: Conceptualization. K.L.: Investigation, formal analysis. J.W.: Supervision, funding acquisition, writing–reviewing, and editing. C.R.: Writing – original draft preparation, data curation, writing – reviewing, and editing. All authors were involved in manuscript editing and approved the version submitted for publication.

## Supporting information

Supporting Information.

## Data Availability

The data that support the findings of this study are available from the corresponding author upon reasonable request.
