# Supplementary material for: Anti‐Hyperglycemic Effects of Oils and Extracts Derived from Sea Buckthorn – A Comprehensive Analysis Utilizing In Vitro and In Vivo Models
Source: Mol Nutr Food Res. 2022 Apr 27;66(12):2101133. doi: 10.1002/mnfr.202101133 (PMC9285508; doi:10.1002/mnfr.202101133)
Supplement: Supplementary file 1 — Supporting Information. [file MNFR-66-0-s001.docx]

Supplemental material to

**Anti-hyperglycemic effects of oils and extracts derived from sea buckthorn - a comprehensive analysis utilizing *in-vitro* and *in-vivo* models.**

*Nicole Ollinger et al.*

**Table S1. HPLC-MS analysis of sea buckthorn oil.** Sea buckthorn oil derived from the geographic region of Zagori was prepared and analyzed by HPLC-MS as described in the main manuscript. Compounds displayed in bold font were reliably identified by comparison with authentic standard.

| **compound name** | **t_R_ (UV)**  **[min]** | **exact mass**  **m/z [MH]^+^** |
| --- | --- | --- |
|  |  |  |
|  | 2,11 | 146,06 |
|  | 2,52 | 127,039 |
|  | 2,96 | 155,0337 |
|  | 3,65 | 127,039 |
|  | 4,3 | 171,0287 |
|  | 6,54 | 138,0548 |
|  | 6,89 | 123,0441 |
|  | 7,88 | 157,0494 |
| isorhamnetin + rhamnose + 2 hexoses | 8,17 | 787,2284 |
|  | 8,36 | 153,0545 |
|  | 9,07 | 183,0651 |
| isorhamnetin + rhamnose + hexose | 9,54 | 625,1761 |
|  | 10,29 | 223,1327 |
|  | 10,5 | 197,1171 |
| isorhamnetin + rhamnose + hexose | 10,57 | 625,1763 |
| isorhamnetin + hexose | 10,88 | 479,1183 |
|  | 11,97 | 265,1433 |
|  | 12,63 | 247,1327 |
| **quercetin** | **12,63** | **303,0497** |
| isorhamnetin + rhamnose | 12,82 | 463,1233 |
|  | 13,1 | 191,1429 |
|  | 13,33 | 209,1535 |
| **isorhamnetin** | **13,93** | **317,0656** |
|  | 15,07 | 181,1222 |
| unidentified compound containing isorhamnetin | 15,97 | 547,1811 |
| unidentified compound containing isorhamnetin | 16,15 | 547,1811 |
|  | 16,52 | 286,1436 |
|  | 17,29 | 271,1691 |
|  | 17,44 | 457,3671 |

**Figure S1. HPLC-MS analysis of sea buckthorn oil.** Upper panel: Chromatogram with PDA detector. Lower panel: Chromatogram with APCI(pos)-MS detector in TIC mode. For methodological details, see main manuscript. The most abundant compound at 13.93 min / 14 min retention time was isorhamnetin.


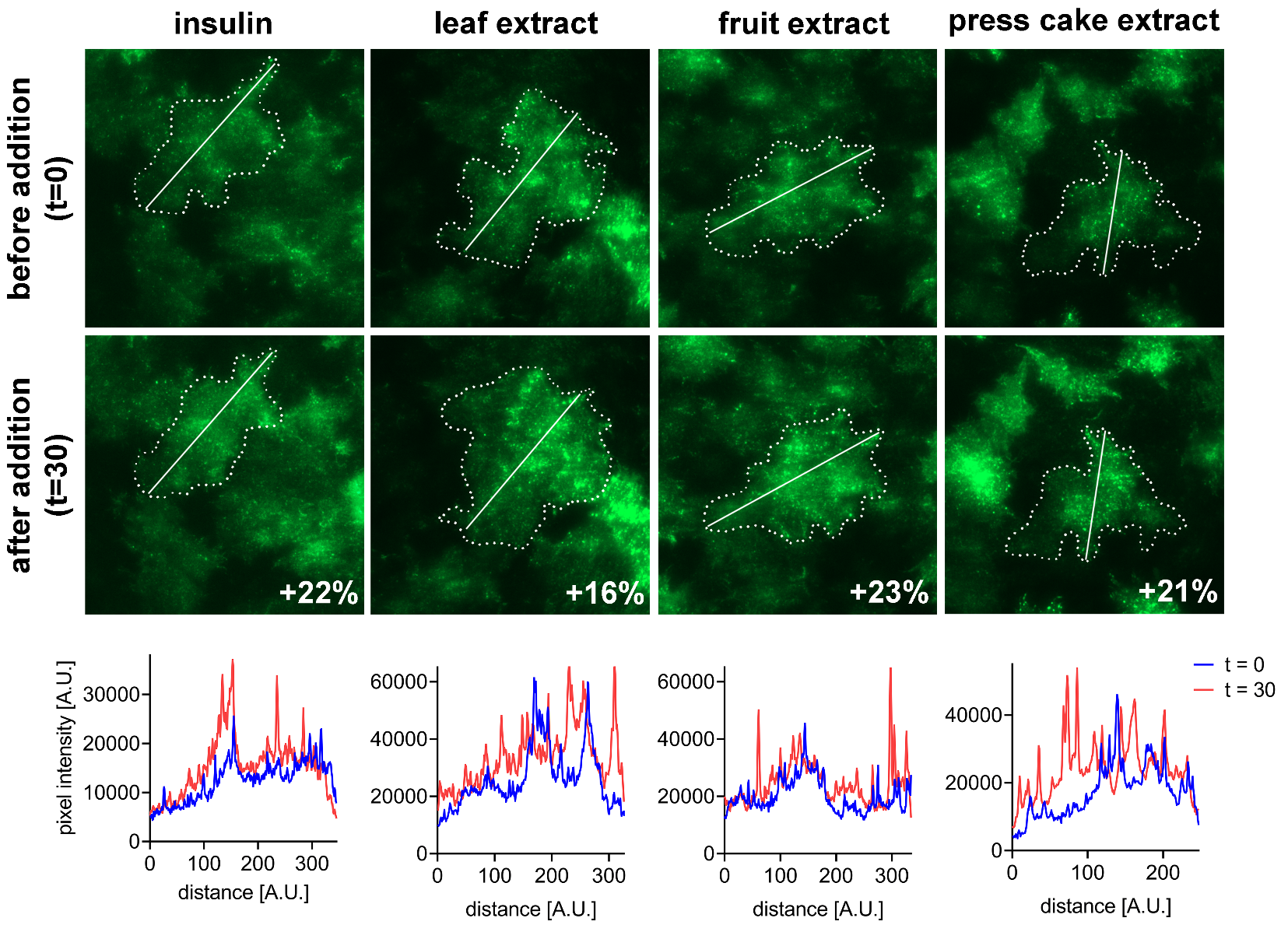


**Figure S2. Sea buckthorn extracts increase GLUT4 cell surface expression.** This figure shows representative images and histogram blots from the experiments presented in figure 3 in the main manuscript. GLUT4-GFP was imaged in TIRF configuration before (upper panel) and 30 min after (middle panel) addition of insulin or sea buckthorn extracts. Numbers in middle panels indicated increases in pixel intensity in cells marked with dashed lines over time. Lower panels show histogram blots along the region marked by the solid white line in the respective upper panels.
